# Supplementary material for: MET variants with activating N‐lobe mutations identified in hereditary papillary renal cell carcinomas still require ligand stimulation
Source: Mol Oncol. 2025 Feb 20;19(8):2366–87. doi: 10.1002/1878-0261.13806 (PMC12330938; doi:10.1002/1878-0261.13806)
Supplement: Supplementary file 1 — Fig. S1. Effect of an anti‐hepatocyte growth factor (HGF) antibody treatment on wound healing induced by HGF on MCF‐7 cells. [file MOL2-19-2366-s002.pdf]

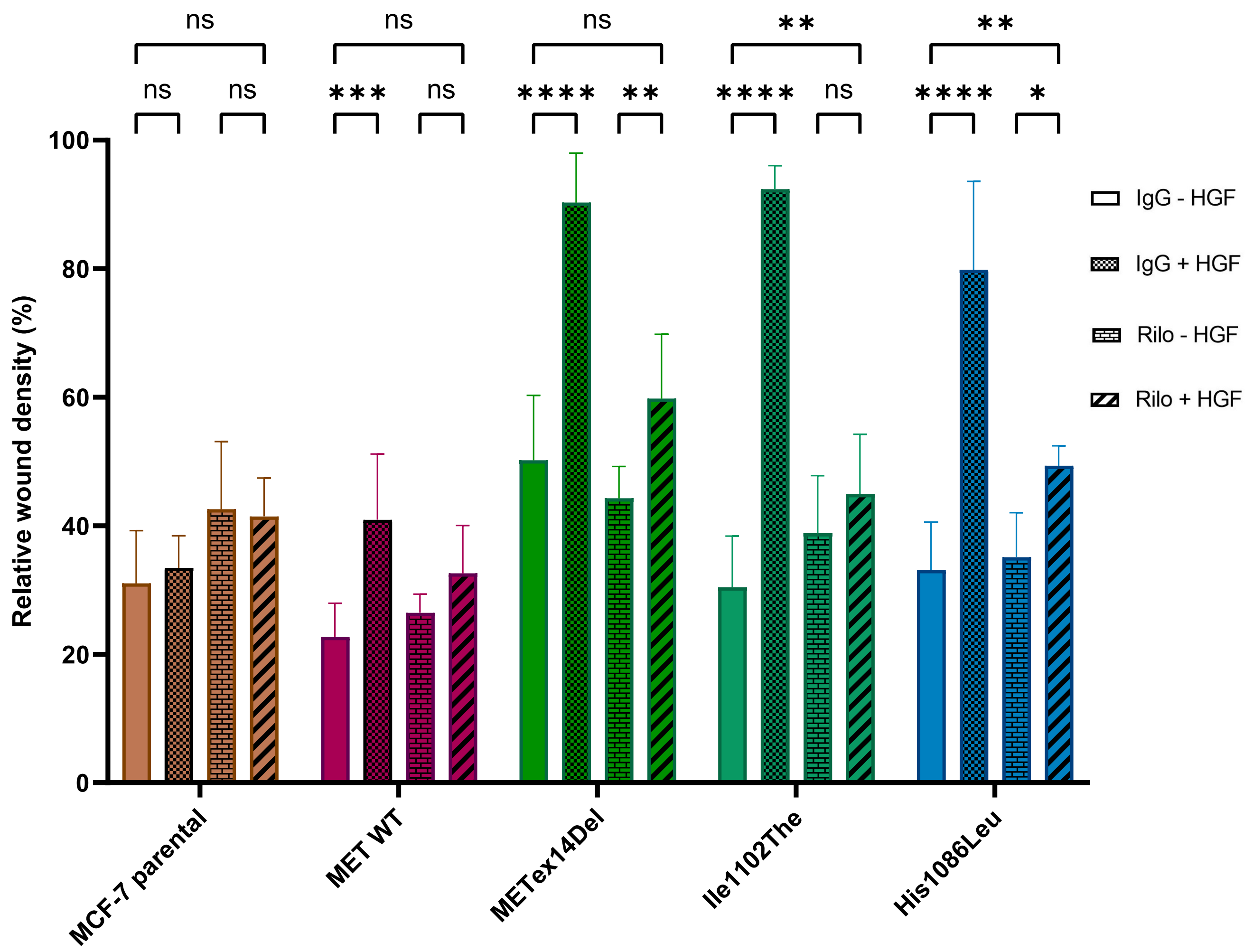

Supplementary Figure S1: **Effect of an anti-HGF antibody treatment on wound healing induced by HGF on MCF-7 cells**. Cells were seeded at 15,000 cells per well in a 96-well plate. 48h later, mitomycin C (10µg/mL) was added for 2h to prevent proliferation. A scratch wound was then performed and cells were stimulated or not with HGF at 30 ng/mL and Rilotumumab (Rilo) or control IgG (IgG) was added at 10µg/ml. Data are expressed as relative wound density 96h after HGF stimulation.n=4; mean ± SEM ; representative of three independent experiments. Statistical analysis by two-way ANOVA p<0.1234 (ns = not significant), p<0.0332 (*), p<0.0021 (**), p<0.0002 (***), p<0.0001 (****).
