# Supplementary material for: MET variants with activating N‐lobe mutations identified in hereditary papillary renal cell carcinomas still require ligand stimulation
Source: Mol Oncol. 2025 Feb 20;19(8):2366–87. doi: 10.1002/1878-0261.13806 (PMC12330938; doi:10.1002/1878-0261.13806)
Supplement: Supplementary file 3 — Fig. S3. Gene enrichment analysis of MET variants under hepatocyte growth factor stimulation. [file MOL2-19-2366-s003.pdf]

A

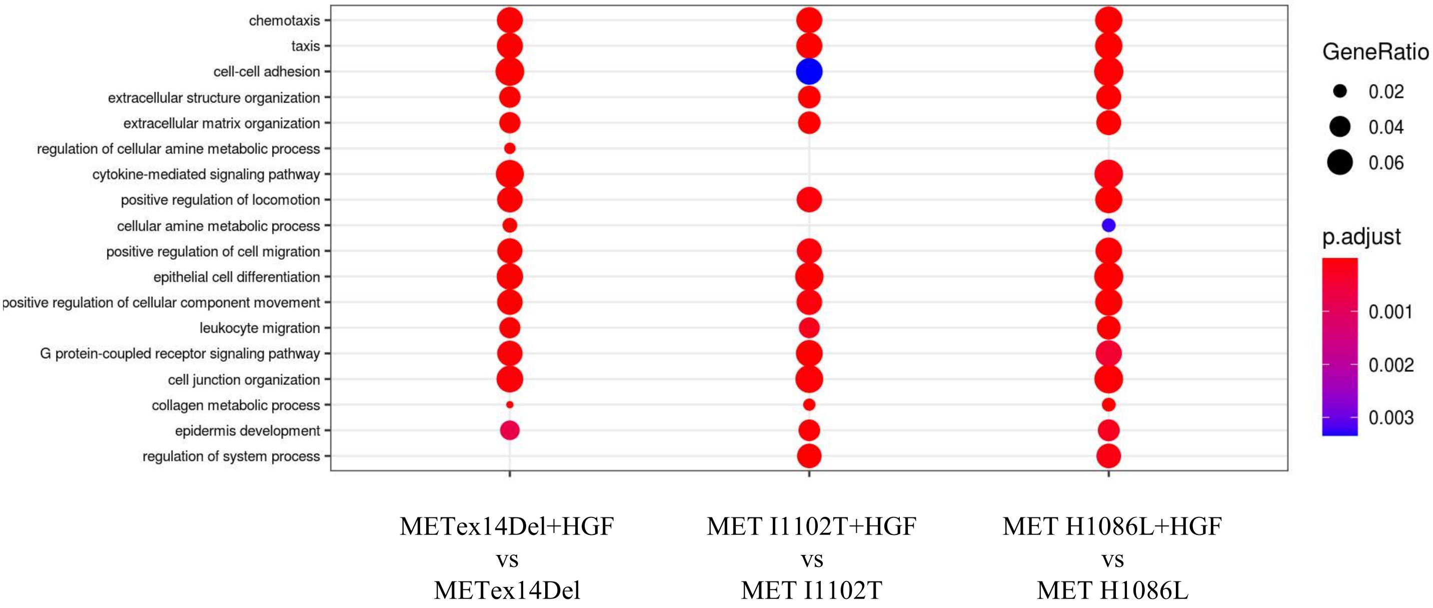

B

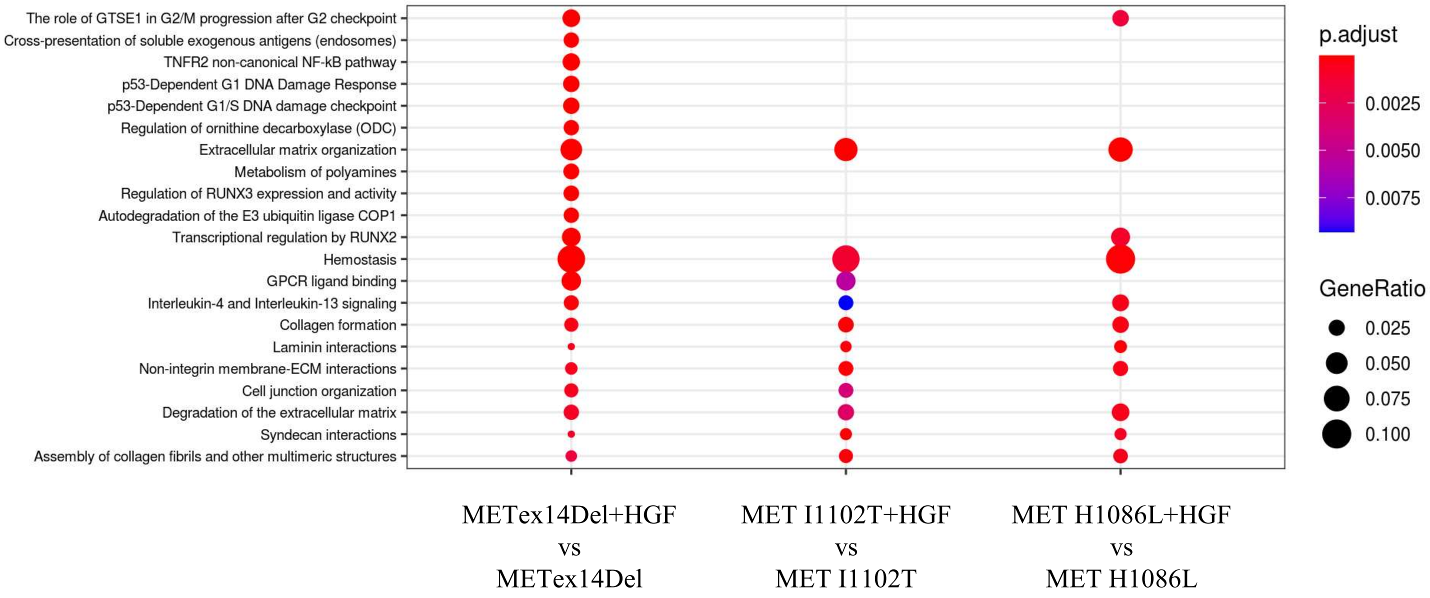

Supplementary Figure S3: **Gene enrichment analysis of MET variants under HGF stimulation.** (A) Dot plot of GO enrichment in 'Biological process' (A) and 'ReactomePA' (B) annotations among genes showing significant differential expression in MCF-7 cells according to whether they were stimulated or not with HGF (p.value adj <0.05 and fold change > 1.5). Dot size represents the gene ratio. Color scale represents the P-value (adjusted).
