# Supplementary material for: MET variants with activating N‐lobe mutations identified in hereditary papillary renal cell carcinomas still require ligand stimulation
Source: Mol Oncol. 2025 Feb 20;19(8):2366–87. doi: 10.1002/1878-0261.13806 (PMC12330938; doi:10.1002/1878-0261.13806)
Supplement: Supplementary file 4 — Fig. S4. Expression of selected genes from the transcriptomic analysis. [file MOL2-19-2366-s006.pdf]

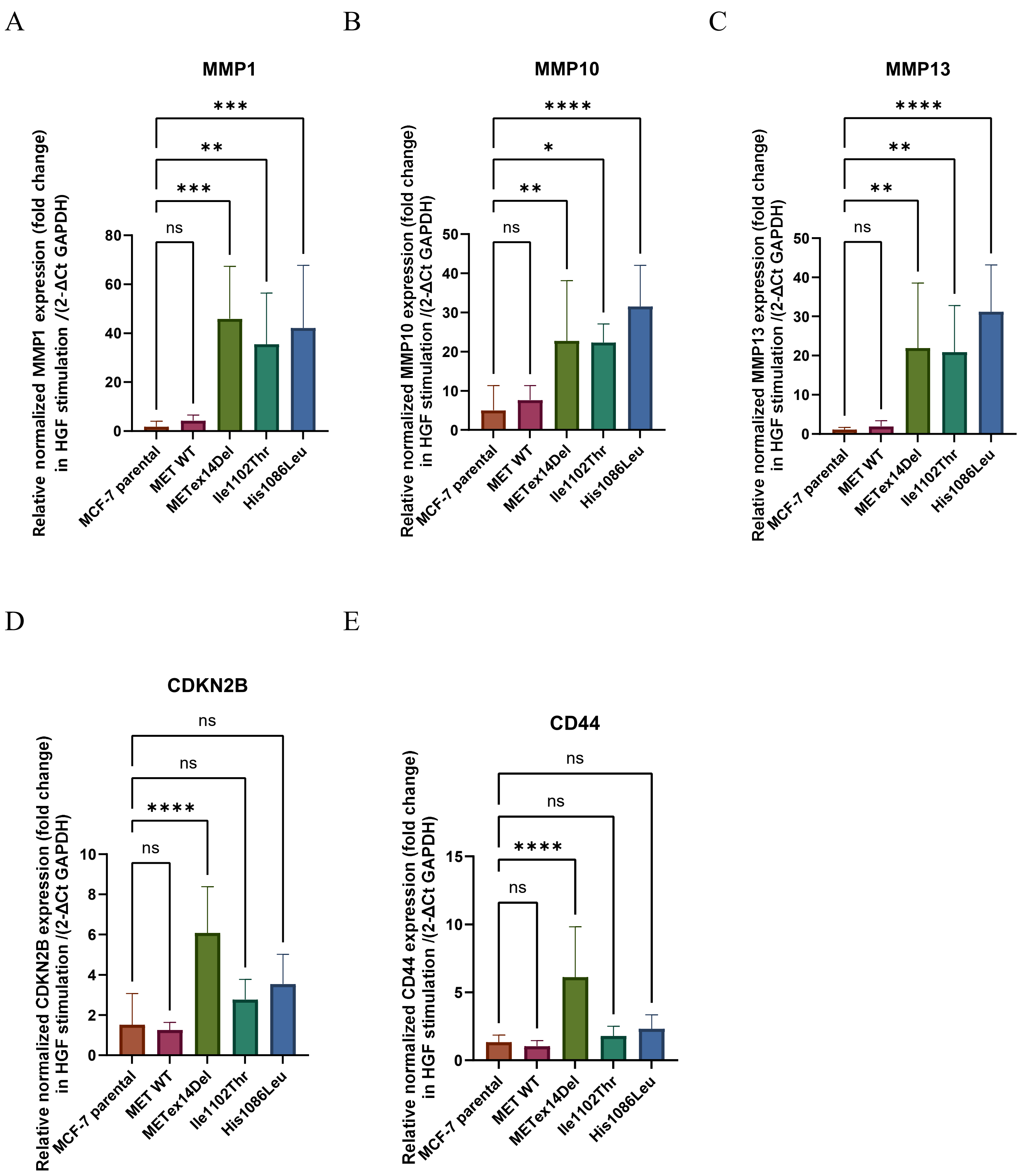

Supplementary Figure S4: **Expression of selected genes from the transcriptomic analysis.** Relative gene expressions to unstimulated conditions of MMP1, MMP10, MMP13, CDKN2A and CD44 determined by RT-qPCR in MCF-7 cells treated or not for 24 h with 30 ng/ml HGF in serum-free medium. * p-value <0.0332; ** p-value <0.0021; *** p-value <0.0002; **** p-value <0.0001.
