# Supplementary material for: MET variants with activating N‐lobe mutations identified in hereditary papillary renal cell carcinomas still require ligand stimulation
Source: Mol Oncol. 2025 Feb 20;19(8):2366–87. doi: 10.1002/1878-0261.13806 (PMC12330938; doi:10.1002/1878-0261.13806)
Supplement: Supplementary file 5 — Fig. S5. MET receptor expression determined by RT‐qPCR in NIH3T3 cells stably transfected with MET variants. [file MOL2-19-2366-s001.pdf]

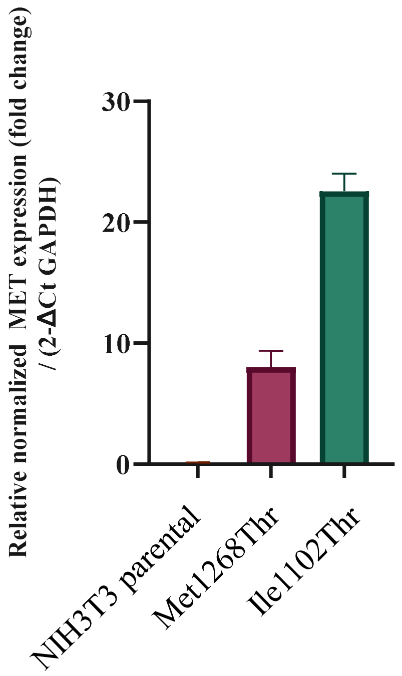

Supplementary Figure S5: MET receptor expression determined by RT-qPCR in NIH3T3 cells stably transfected with MET variants. Level of MET expression was determined by RT-qPCR in parental NIH3T3 and cells stably transfected with MET Met1268Thr and MET Ile1102Thr .
